# Knowledge of Oral Health in Pregnant and Puerperal Women: Effect of a Short Educational Intervention

**DOI:** 10.3390/dj14100683

**Published:** 2026-10-19

**Authors:** Alicia Aznar-Marín, María Dolores Casaña-Ruiz, Beatriz Marcos-Puig, María Ángeles Velló-Ribes, Alfredo Perales-Marín, Montserrat Catalá-Pizarro

**Affiliations:** 1 Department of Stomatology, Faculty of Medicine and Dentistry, University of Valencia, 46010 Valencia, Spain; aznma2@alumni.uv.es (A.A.-M.); m.angeles.vello@uv.es (M.Á.V.-R.); montserrat.catala@uv.es (M.C.-P.); 2 Department of Obstetrics and Gynecology, La Fe University and Polytechnic Hospital, 46026 Valencia, Spain; marcos_bea@gva.es (B.M.-P.); alfredo.perales@uv.es (A.P.-M.); 3 Department of Pediatrics, Obstetrics and Gynecology, Faculty of Medicine and Dentistry, University of Valencia, 12006 Valencia, Spain

**Keywords:** oral health, pregnancy, puerperal women, health education, maternal and child health, knowledge

## Abstract

**Background/Objectives:** Pregnancy and the postnatal period offer an opportunity to establish preventive oral health behaviors benefiting mothers and children. However, many pregnant women have limited knowledge of oral health and early childhood caries prevention. This study assessed oral health knowledge among pregnant and puerperal women attending a tertiary referral hospital in Spain and evaluated the effect of a brief educational intervention. **Methods:** A quasi-experimental pre–post study was conducted following TREND guidelines and registered at ClinicalTrials.gov (NCT07413393). Participants completed a questionnaire on demographics, oral health knowledge, attitudes, and educational background. They then received an educational intervention based solely on written educational materials, consisting of a brief explanation and a leaflet based on international paediatric dentistry guidelines. Knowledge changes among women completing follow-up were analyzed using the paired Student’s *t*-test and Wilcoxon signed-rank test. **Results:** Of 229 women completing the baseline assessment, 130 completed follow-up. Educational level was the main independent factor associated with higher baseline knowledge. Major gaps concerned maternal influence on early oral microbial colonization, recognized by only 38.86% of participants, and recognition of white spot lesions as the earliest sign of caries (10.77%). Overall knowledge improved significantly after the intervention (*p* = 0.018), particularly regarding nocturnal bottle feeding and caries (*p* = 0.0001) and timing of the first dental visit (*p* = 0.04). **Conclusions:** Pregnant and puerperal women showed important gaps in preventive oral health knowledge. A brief educational intervention improved overall knowledge, although deficiencies remained. Integrated multidisciplinary oral health education during prenatal care may improve maternal and child outcomes.

## 1. Introduction

During pregnancy, women experience hormonal and physiological changes that may affect oral tissues and increase susceptibility to oral diseases. Oral health problems are common during pregnancy and may become more frequent as pregnancy advances [1,2]. Hormonal fluctuations may affect the teeth, supporting tissues, and oral mucosa and, when combined with dietary changes and inadequate oral hygiene practices, may increase the risk of dental caries and periodontal disease [2,3].

Periodontal disease during pregnancy has been associated with adverse maternal and neonatal outcomes, including preterm birth, low birth weight, preeclampsia, and gestational diabetes [4]. In addition, pregnancy-related dietary changes, together with nausea and gastric reflux, may contribute to an increased risk of dental caries by promoting frequent sugar intake and enamel erosion. Delayed dental care and the lack of routine oral examinations may further increase the risk of oral diseases during this period [5]. Despite being largely preventable, ECC remains the most prevalent chronic disease in childhood, affecting more than 600 million children worldwide [6]. Compared with dental caries in older children and adults, ECC is characterized by its rapid progression, multifactorial etiology, and specific preventive and management approaches [6].

The World Health Organization recognizes the importance of adopting preventive strategies throughout the life course, beginning in the preconception, prenatal, and postnatal periods [7]. Pregnancy is considered a particularly valuable window of opportunity for promoting oral health and establishing healthy behaviors that may benefit both mothers and their children. Healthcare professionals involved in prenatal care, including physicians, midwives, and nurses, play an important role in promoting healthy habits, providing oral health education, and facilitating referral to dental services when needed [7]. Educational interventions during pregnancy have been shown to improve maternal knowledge and promote more favorable attitudes and behaviors related to child oral health [8].

However, several studies have shown that pregnant women continue to have limited knowledge and awareness regarding oral health during pregnancy and early childhood oral health prevention. Persistent misconceptions about the safety of dental care, the impact of maternal oral health on pregnancy outcomes, and preventive practices for infants may negatively influence oral health behaviors and healthcare utilization [9,10,11]. Although educational interventions have demonstrated beneficial effects, important knowledge gaps remain, particularly regarding key preventive concepts. Therefore, assessing maternal knowledge and evaluating practical educational approaches may contribute to improving oral health promotion during pregnancy.

In this context, the aim of the present study was to assess oral health knowledge among pregnant and puerperal women and to evaluate the effect of a brief educational intervention on improving such knowledge.

## 2. Materials and Methods

Prior to study initiation, ethical approval was obtained from the Research Ethics Committee for Medicines (CEIm) of Hospital Universitari i Politècnic La Fe, in accordance with the principles of the Declaration of Helsinki and current regulations on personal data protection. Ethical approval was granted on 22 December 2021 (approval number: 2021-784-1).

A quasi-experimental pre–post intervention study was conducted at a tertiary-care hospital in an Autonomous Community in eastern Spain, following the Transparent Reporting of Evaluations with Nonrandomized Designs (TREND) guidelines [12]. The study protocol was also registered at ClinicalTrials.gov (Protocol Registration and Results System, NCT07413393) https://register.clinicaltrials.gov/ (accessed on 29 July 2026).

The study population consisted of pregnant and puerperal women prior to hospital discharge. Participants included women who completed only the baseline questionnaire and women who received the educational leaflet and completed both the baseline and post-intervention questionnaires. The minimum required sample size was estimated based on an annual average of 3.500 deliveries at the study center. Assuming a non-response rate of 20%, a minimum sample of 190 pregnant women was calculated using the finite population sampling formula, with a 95% confidence level and a 10% margin of error.

Inclusion criteria:

- Pregnant and puerperal women aged 18–45 years with vaginal delivery or cesarean section of healthy newborn.
- Ability to understand Spanish.

Exclusion criteria:

- Pregnancy- or delivery-related complications.
- Newborns with major congenital malformations.
- Lack of informed consent.

A questionnaire was developed based on a literature review, previously published questionnaires used in related studies [13,14,15,16,17], and expert opinions regarding oral healthcare during pregnancy. The baseline questionnaire (Supplementary Files S1 and S2) consisted of 45 items divided into four sections: demographic characteristics (*n* = 14), oral health knowledge (*n* = 14), attitudes and practices (*n* = 12), and educational background (*n* = 5). The maximum possible score for the oral health knowledge section was 28 points.

Even though the initial questionnaire collected general data on maternal attitudes and practices, the scope of this study was intentionally limited to assessing knowledge of oral health, in order to focus specifically on the immediate outcomes of the educational intervention.

Knowledge items were scored as follows: correct answer (2 points), incorrect answer (0 points), and “I do not know” response (1 point). Levels of knowledge were categorized according to quartiles as low (25th percentile), moderate (50th percentile), and high (75th percentile).

Before the main study, a pilot study was conducted to evaluate questionnaire comprehensibility, wording adequacy, and estimated completion time.

Thirty pregnant women who were independent of the study setting and were not included in the main study participated in the pilot phase. This step made it possible to identify editing errors, inconsistencies, and comprehension issues. Consequently, two questionnaire items were reworded, one in the knowledge section and one in the attitudes section.

An educational leaflet was developed according to recommendations from major international and national paediatric dentistry organizations, including the American Academy of Pediatric Dentistry (AAPD) [18], the European Academy of Paediatric Dentistry (EAPD) [19], the Latin American Association of Pediatric Dentistry (ALOP) [3], and the Spanish Society of Paediatric Dentistry (SEOP) [20]. To ensure clarity and reproducibility, the terminology was specifically adapted to be clear, culturally appropriate, and understandable for the target population, avoiding complex medical jargon. The content was reviewed and verified by two experts in paediatric dentistry to guarantee scientific accuracy. The leaflet was organized into two main sections: “For Mothers” and “For Your Baby”, summarized in Table 1.

**Figure 1:**
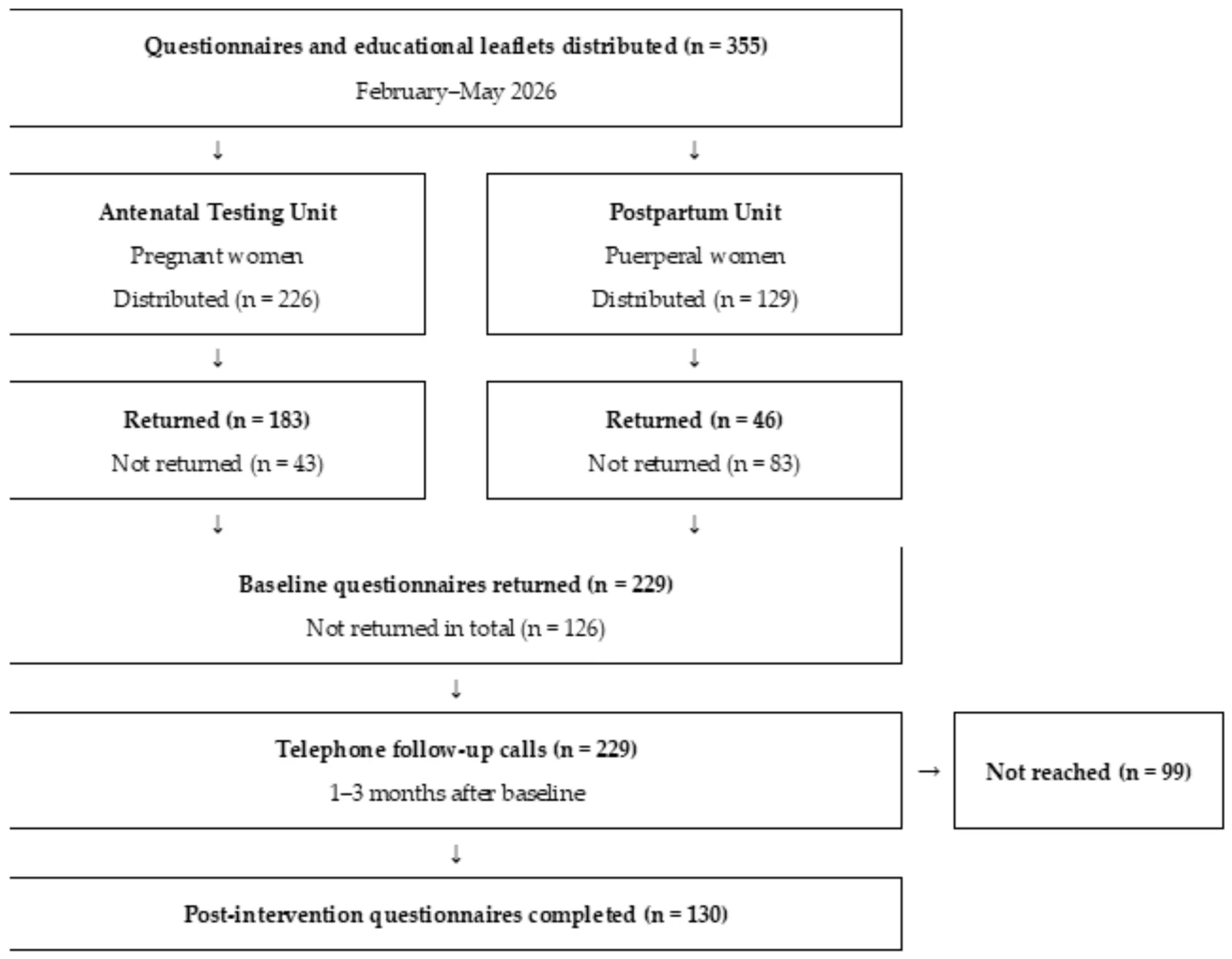
Flowchart of the survey process. Educational leaflets were distributed individually to participants immediately upon completion of the questionnaire.

Between February 2026 and May 2026, baseline questionnaires were administered to pregnant women during their attendance at the fetal monitoring unit and puerperal women who were hospitalized. Participants received information regarding the study objectives, voluntary participation, and the requirement to provide written informed consent before enrollment. The investigator remained available during questionnaire completion to resolve questions and address any issues that arose. During the study period, all women who had completed the baseline questionnaire received the educational leaflet. Follow-up assessment was subsequently performed during the postpartum period using the post-intervention questionnaire. While initial questionnaire distribution and leaflet delivery at the Day Hospital and inpatient ward began during the first months of the year, the telephone call phase for postpartum re-evaluation was initiated in April 2026. This timing ensured that women enrolled at the end of their pregnancy (weeks 38–40) had already completed the critical immediate postpartum period. Post-intervention questionnaires were administered by telephone between one and three months after the initial questionnaire, using a mobile phone exclusively dedicated to the study and managed by the hospital (Figure 1). During each call, responses were recorded directly into electronic forms by the principal investigator.

Data obtained from the different study groups were entered into an Excel^®^ (v2607, Microsoft Corporation, Redmond, WA, USA, EE. UU., 2026) spreadsheet and analyzed using IBM SPSS^®^ Statistics for Windows v26.0 (IBM Corp.: Armonk, NY, USA; 2019) and MedCalc^®^ Statistical Software, v23.4, MedCalc Software Ltd., Ostende, Belgium, 2025.

A descriptive analysis of demographic characteristics, educational background, and oral health knowledge was performed. Means and 95% confidence intervals were calculated for non-categorical variables.

For comparative analyses, reference groups included Spanish women, women with university education, paid employment, a partner, previous children, follow-up in public healthcare centers, and pregnancy-related complications. Associations between oral health knowledge and demographic variables, and educational background were analyzed using Student’s *t*-test.

To evaluate the effect of the educational intervention, the paired Student’s *t*-test and the non-parametric Wilcoxon signed-rank test were applied. Odds ratios (ORs) and logistic regression analyses were used to assess associations between variables.

A logistic regression model was applied, with the dependent variable being the overall level of knowledge, estimated as 1 based on the 50th and 75th percentiles. The independent variables included in the model were the epidemiological variables statistically associated with knowledge, as well as those with a potential explanatory association supported by the literature. To assess the validity and statistical calibration of the logistic regression models generated, the Hosmer–Lemeshow test was performed.

A *p*-value < 0.05 was considered statistically significant.

## 3. Results

### 3.1. Demographic Characteristics

A total of 229 women completed the baseline questionnaire, corresponding to a response rate of 64.50%. Follow-up assessment was completed by 130 women (response rate: 56.77%). The mean age of participants in the follow-up group was 33.21 ± 6.46 years. Of these, 18.14% were foreign-born, most had a partner (92.54%), and participants were almost equally distributed between primiparous (52.63%) and multiparous (47.37%) women. Regarding newborn characteristics, 44% were girls and 56% were boys, with a mean birth weight of 3.31 kg and a mean gestational duration of 276.3 days.

Overall, 43.81% of participants did not have university education, 72.89% had paid employment, and 64.32% received prenatal follow-up in public healthcare centers. Among women with previous children, 84.26% reported that their other children had no history of dental caries (Table 2).

### 3.2. Oral Health Knowledge at Baseline Assessment

Among the 14 knowledge questions included in the questionnaire (maximum score: 28 points), the mean score was 19.58 ± 3.92 points. Most participants (78.17%) correctly identified the bacterial implication in dental caries; however, only 38.86% of participants understood how maternal oral health impacts initial oral microbial acquisition in children. In addition, 69% were aware that oral problems affecting primary teeth may also affect permanent dentition. Regarding preventive practices, 43.23% reported that toothbrushing should begin with the eruption of the first tooth. Most women (84.72%) knew that sharing utensils or cleaning a pacifier with the mouth should be avoided. Although 65.07% recognized the preventive role of fluoride, only 24.45% considered its use appropriate before 3 years of age.

Knowledge gaps were also identified regarding early signs of dental caries and preventive dental visits. In the overall baseline sample (N = 229), only 10.04% of participants recognized white spot lesions as the earliest sign of dental caries, and 41.05% correctly identified that the first dental visit should take place by 12 months of age (Supplementary File S3).

A logistic regression was performed to evaluate knowledge levels based on the 75th (high) and 50th (moderate) quartiles; no significant results were obtained. After restricting the model to key sociodemographic variables: age, place of birth, marital status, previous children, level of education, and complications during pregnancy, educational level and having previous children were identified as independent factors associated with knowledge, both in the 75th quartile (OR= 3.5, 95% CI: 1.5–8.14, *p*= 0.004 and OR= 3.41, 95% CI: 1.68–6.94, *p* = 0.0007, respectively) as well as at the 50th (OR = 1.94, 95% CI: 1.05–3.60, *p* = 0.04 and 1.99, 95% CI: 1.11–3.55 *p* = 0.02, respectively). Both models showed adequate calibration based on the Hosmer–Lemeshow test (*χ*^2^ = 12.88, DF 8 and *p* = 0.116 and *χ*^2^ =6.04; DF 8 and *p* = 0.64, respectively).

### 3.3. Effect of the Educational Intervention on Oral Health Knowledge

To evaluate the effect of the educational intervention, participants were categorized according to baseline knowledge score quartiles as low (Q1 ≤ 18), intermediate (Q1–Q3: 19–22), and high (≥23), and the (calculated) median score was 20 points. The same thresholds were applied to both baseline and post-intervention assessments to allow direct comparison.

To evaluate the impact of the educational intervention, pre- and post-intervention scores were compared. To maintain focus on key outcomes and avoid redundancy, detailed pre- and post-assessment data are presented only for variables that demonstrated statistically significant changes (*p* < 0.05).

**Figure 2:**
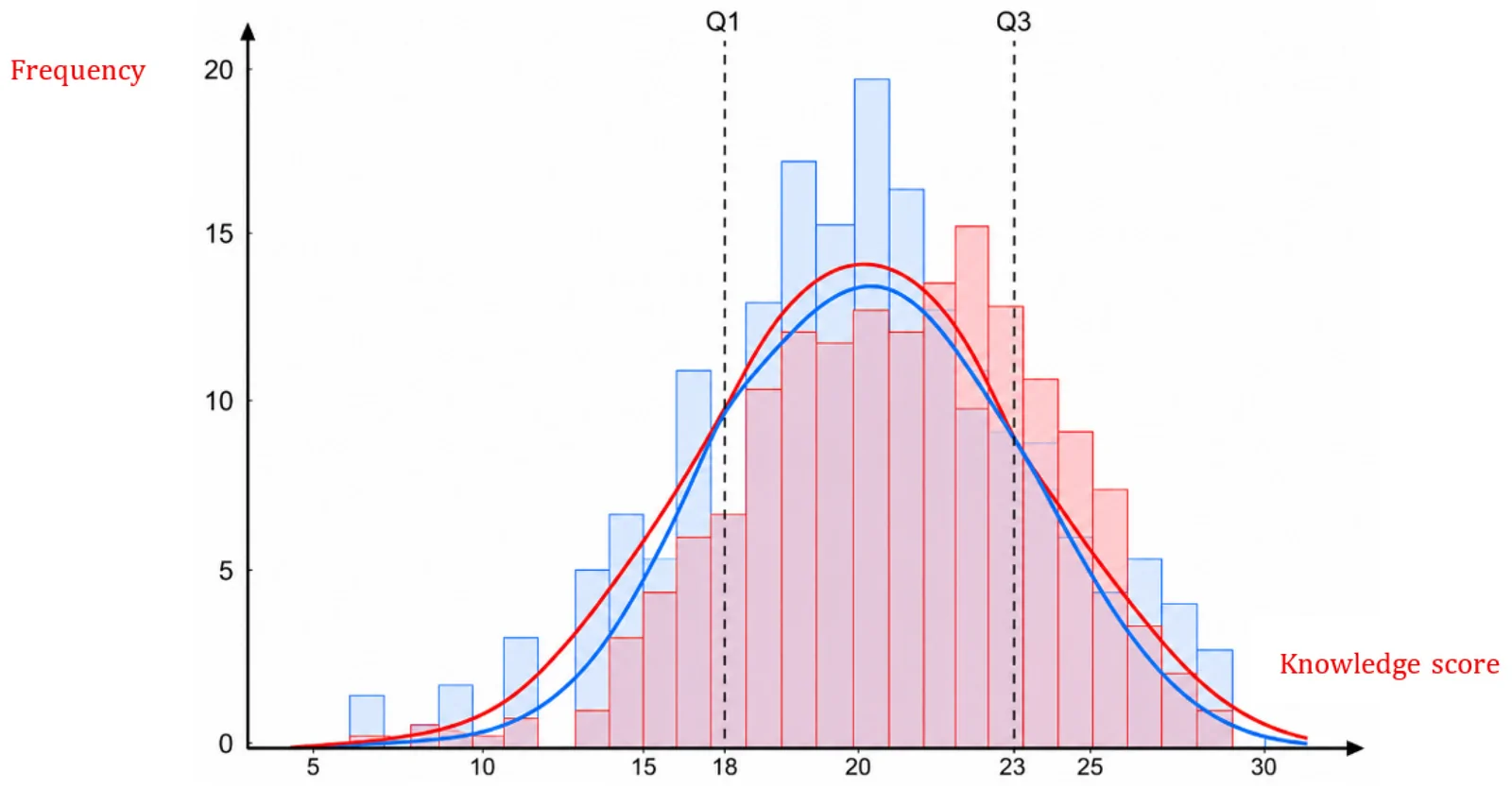
Knowledge score distribution before and after the educational intervention (*n* = 130). The *X*-axis represents the total knowledge scores obtained by pregnant women, categorized by quartiles. The *Y*-axis represents the frequency of participants achieving each score or score range. Dashed vertical lines delimit the first quartile (Q1 = 18) and third quartile (Q3 = 23). Comparing the pre-intervention distribution (blue curve) with the post-intervention distribution (red curve) reveals a clear rightward shift in participants’ scores.

At baseline (N = 130), 46 participants were classified within the lowest quartile, 29 within the second quartile, 32 within the third quartile, and 23 above the third quartile. Following the educational intervention, the distribution of scores changed, with fewer participants in the lowest quartiles (*n* = 61), and a greater number in the third quartile (*n* = 44) and highest category (*n* = 25) (Figure 2).

Paired Student’s *t*-test demonstrated a statistically significant improvement in oral health knowledge after the intervention (mean difference = −0.754; t = −2.396; *p* = 0.018). As the difference was calculated as baseline score minus post-intervention score, the negative mean difference indicates that post-intervention scores were, on average, higher than baseline scores, supporting an overall increase in knowledge. These findings were confirmed by the Wilcoxon signed-rank test (Z = −2.254, *p* = 0.024), indicating that a substantial proportion of participants (*n* = 69) improved their knowledge scores following the educational intervention.

**Figure 3:**
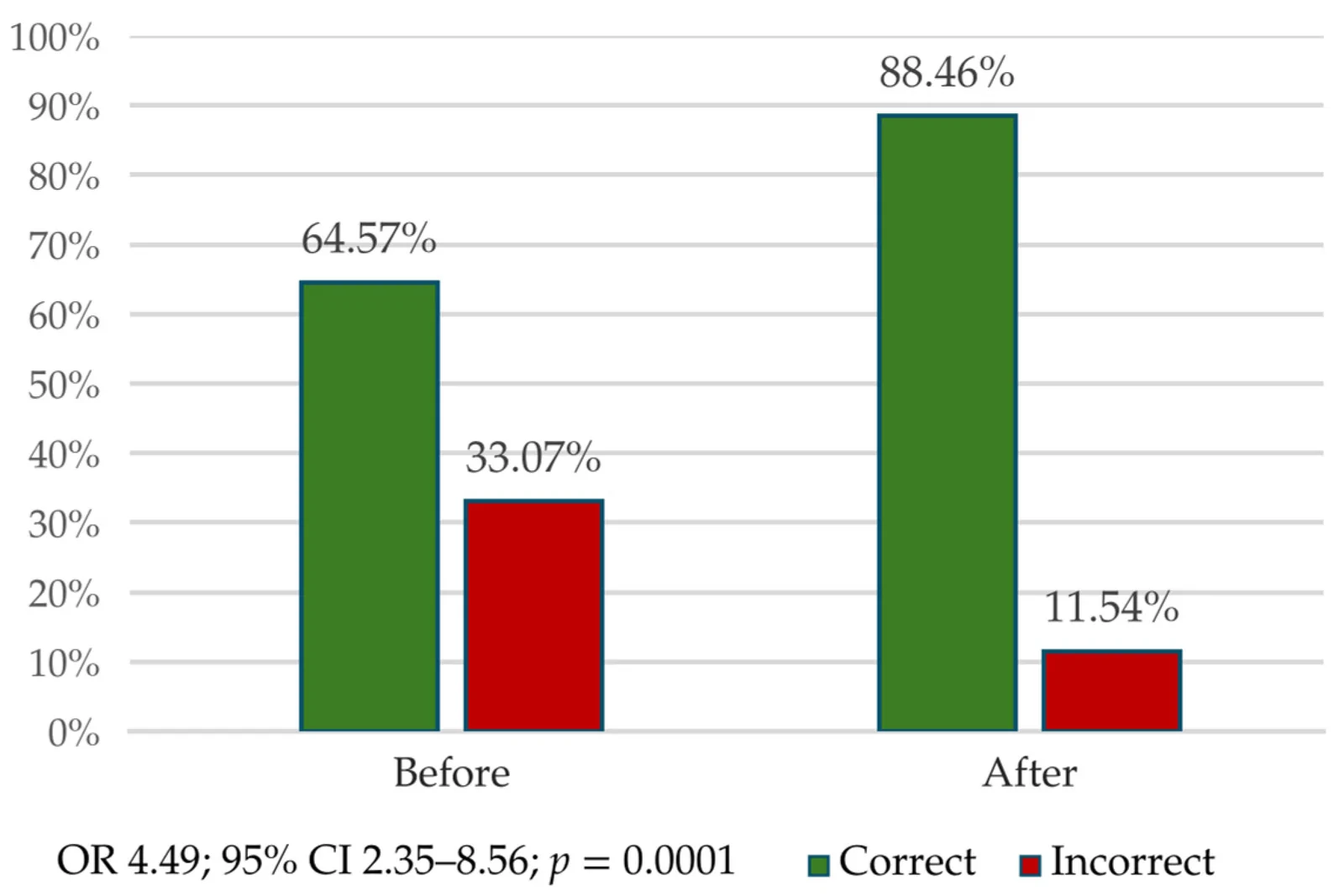
Before-and-after comparison: “bedtime bottle use containing milk or juice and its association with tooth decay, even in children aged 2–3 years”.

When individual knowledge items were analyzed, significant improvements were observed in several preventive oral health concepts. Knowledge regarding the association between nocturnal bottle feeding with milk or juice and dental caries significantly improved after the intervention (OR = 4.49, 95% CI: 2.35–8.56, *p* = 0.0001), with correct responses increasing from 82 to 115 participants, while incorrect responses decreased from 45 to 15 (Figure 3).

**Figure 4:**
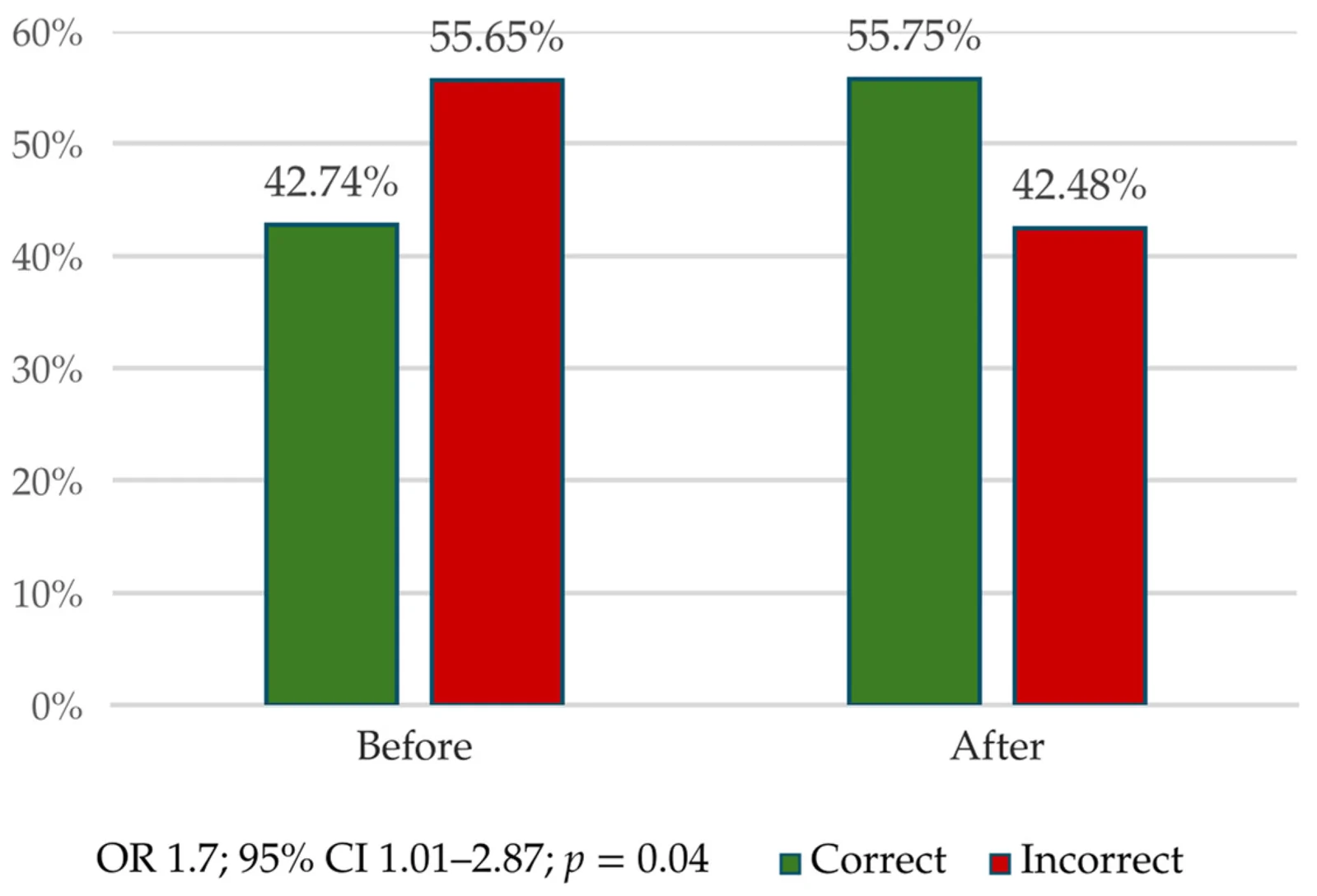
Pre- and post-intervention responses regarding the timing of a child’s first dental visit.

Similarly, knowledge regarding the recommended timing of the child’s first dental visit showed a significant improvement after the intervention (OR = 1.7, 95% CI: 1.01–2.87, *p* = 0.04) (Figure 4).

**Figure 5:**
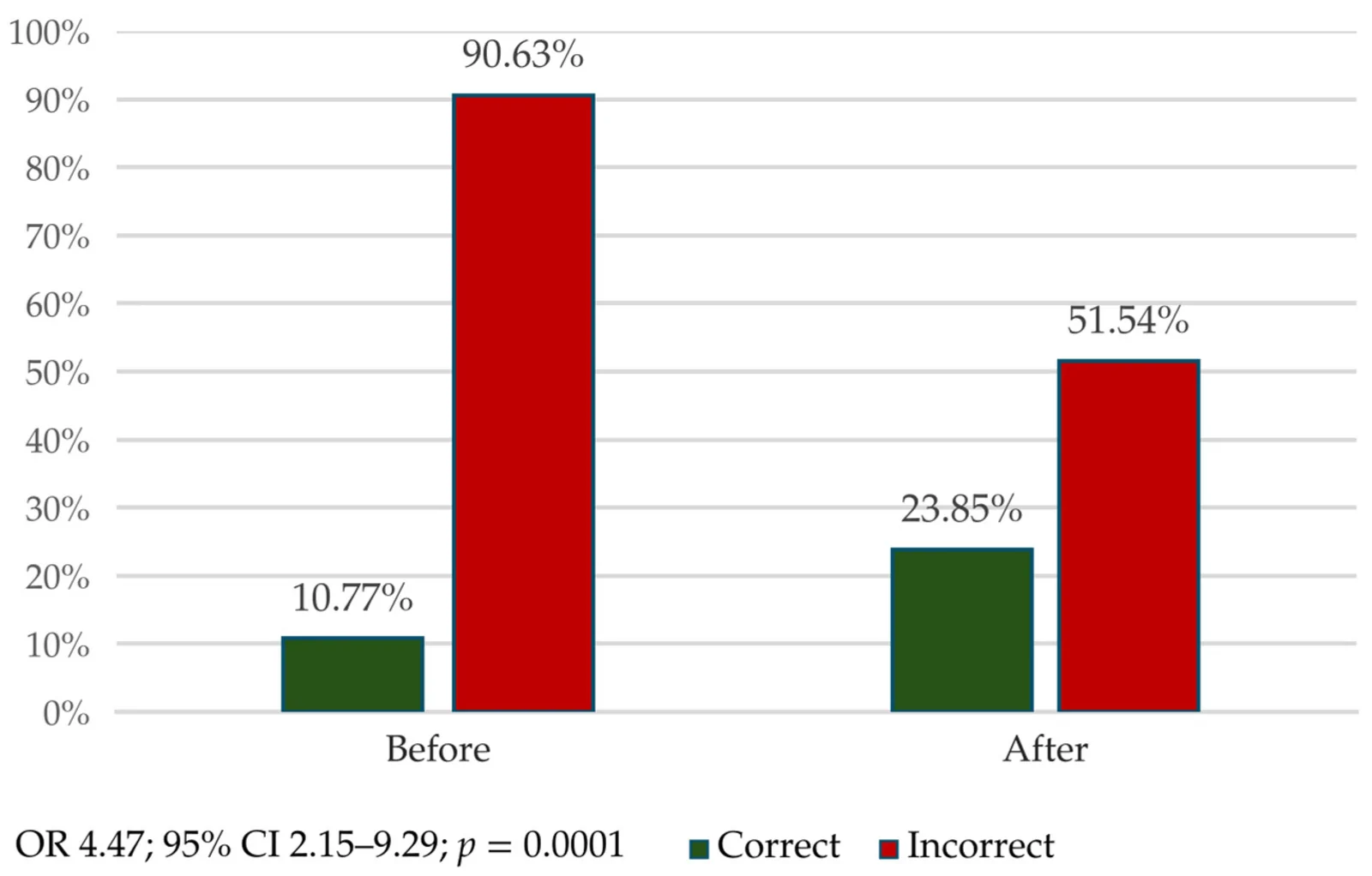
Pre- and post-intervention comparison of responses to the question: ‘Is a white spot typically the first sign of dental caries’?

Among participants who completed the follow-up assessment (*n* = 130), recognition of white spot lesions as the earliest sign of dental caries increased significantly following the intervention (OR = 4.47, 95% CI: 2.15–9.29, *p* = 0.0001), with correct responses rising from 14 participants (10.77% pre-intervention baseline for this sub-sample) to 31 participants (23.85% post-intervention) (Figure 5).

In contrast, knowledge regarding hormonal changes affecting gingival health showed lower rates of correct responses after the intervention.

No statistically significant differences were observed for the remaining knowledge variables after odds ratio analysis.

## 4. Discussion

The present study found that pregnant and puerperal women exhibited a moderate level of oral health knowledge, with a baseline mean score of 19.58 ± 3.92 out of a maximum of 28 points. Although this result suggests a reasonable degree of awareness regarding oral health during pregnancy, important deficiencies were identified in several key areas related to maternal and child oral health. These findings are consistent with previous studies conducted in different populations, which have generally reported low to moderate levels of oral health knowledge among pregnant women despite increasing awareness of the importance of oral health during pregnancy [21,22,23].

In our study, educational level and parity emerged as the main factors associated with oral health knowledge. Women with higher educational levels achieved significantly higher scores, supporting the findings reported by Abiola et al. [24], Barbieri et al. [25], Desai et al. [26], Chawłowska et al. [27], Wassihun et al. [28], Niazi et al. [29], Llena et al. [30], Bhandari et al. [31] and Motta et al. [32]. In a multivariate evaluation of pregnant women, Bhandari et al. [31] identified educational status as the single sociodemographic variable independently associated with oral health knowledge, attitudes, and practices (*p* < 0.001), while observing that 79.4% of pregnant women lacked prior professional guidance on oral health. Similarly, Motta et al. [32] demonstrated that low maternal knowledge (60.4%) was significantly conditioned by lower education levels and multiparity (*p* < 0.05). In addition, the current literature highlights the influence of health literacy on intervention effectiveness. In line with our findings, where educational level was associated with higher baseline knowledge, Vilella et al. [33] identified oral health literacy as a key predictor of both knowledge acquisition and retention, while Bansal et al. [13] also reported greater improvements among women with higher educational attainment. A higher educational level may facilitate access to health information, improve comprehension of preventive messages, and enhance the ability to seek out and critically appraise health-related information [28,29,30,34,35].

The relationship between parity and oral health knowledge remains controversial. Our results demonstrated higher knowledge levels among multiparous women, consistent with studies suggesting that previous pregnancies and repeated contact with healthcare professionals may increase oral health knowledge [25,26,27,28,29,30,34,35]. Cagetti et al. [36] observed that maternal experience gained from raising multiple children positively influenced oral health knowledge scores (*p* = 0.04), even though 73.17% of surveyed mothers received no advice regarding maternal or infant oral health during prenatal care. Collectively, these findings underscore that while parity plays a contextual role, baseline health literacy remains constrained across diverse populations in the absence of structured, equitable prenatal educational outreach. Conversely, Llena et al. [30] and Lakshmi et al. [37] reported higher knowledge levels among primiparous women, whereas Wassihun et al. [28] found no significant association between parity and oral health knowledge. These inconsistent findings suggest that oral health education should be systematically provided to all pregnant women, regardless of parity.

Other sociodemographic factors were also associated with oral health knowledge. Employment status showed a significant relationship with knowledge levels, possibly reflecting differences in socioeconomic circumstances, access to health information, and opportunities for interaction with healthcare services. Associations between employment status and oral health knowledge have also been reported in previous studies [30,34]. In the Spanish study conducted by Llena et al. [30], nationality was independently associated with general oral health knowledge, whereas the association with employment status did not remain significant after adjustment for other sociodemographic factors. Therefore, differences according to employment status and nationality may reflect interrelated disparities in educational opportunities, cultural beliefs, health literacy, and access to preventive healthcare services.

Overall, our findings suggest that educational attainment is the strongest determinant of maternal oral health knowledge, although employment status, and broader social and cultural factors also appear to influence knowledge acquisition during pregnancy.

Although the overall level of knowledge was moderate, several important gaps were identified in topics directly related to maternal and child oral health. These deficiencies are clinically relevant because they concern preventive behaviors that may influence both maternal oral health and the future oral health of the child.

Regarding maternal oral health and adverse pregnancy outcomes, just half of the participants were aware that oral health problems during pregnancy could affect the baby. Although this proportion is comparable to that reported by Chawla et al. [38] and Gaszynska et al. [39] and higher than in several previous studies [30,35,38,40], it nevertheless indicates that a substantial proportion of women remain unaware of the potential consequences of poor maternal oral health on pregnancy and fetal outcomes. Knowledge of the association between periodontal disease and adverse pregnancy outcomes remains particularly limited worldwide, despite growing evidence linking periodontal inflammation to complications such as preterm birth, low birth weight, and preeclampsia [24,30,41,42,43].

One of the most notable findings was the limited baseline awareness regarding the maternal influence on early bacterial colonization. Initially, although most participants (78.17%) identified dental caries as a microorganism-related condition, only 38.89% recognized that cariogenic bacteria could be acquired by the infant from the mother. Similar baseline deficiencies have been reported by Barbieri et al. [25], Chawłowska et al. [27] and Gaffar et al. [40]. Following the intervention, awareness of this early microbial exposure increased to 52.67%. Under the framework of the current ecological plaque hypothesis, maternal transmission of microorganisms—particularly *Streptococcus mutans*—is understood not as the direct transmission of a contagious disease, but as a key factor shaping the initial assembly and ecological balance of the infant’s oral microbiome [18,44,45]. Consequently, early colonization by cariogenic bacteria, when combined with frequent exposure to dietary sugars, host susceptibility, and environmental factors, creates a dysbiotic environment that drives early childhood caries. These findings highlight that educational interventions should not only explain early microbial acquisition but also emphasize managing the broader oral ecosystem and dietary habits.

Important gaps were also observed in knowledge related to the prevention of early childhood caries. Although most participants recognized the harmful effects of nocturnal bottle feeding, consistent with previous studies [41,46], misconceptions persisted regarding the appropriate timing of the first dental visit, fluoride use in young children, and the initiation of oral hygiene practices. Similar deficiencies have been reported internationally and suggest that preventive recommendations are not being consistently communicated to expectant mothers [27,34,37,38,41].

Another particularly striking finding was the remarkably low baseline recognition of white spot lesions as the earliest sign of dental caries (10.77% overall and 24.44% in the follow-up cohort). Although our written intervention produced a statistically significant improvement, nearly three-quarters of participants remained unable to identify this early sign. Early identification of these non-cavitated lesions is clinically vital in early childhood caries (ECC) management to enable secondary preventive measures, such as remineralization protocols, before irreversible cavitation occurs [7]. This finding aligns with observations by Angarita-Díaz et al. [47], who evaluated a neuroeducational strategy in caregivers and noted that, despite immediate gains through practical demonstrations, the recognition of white spot lesions was particularly vulnerable to knowledge decay at six-month follow-up. Taken together, these evidence streams highlight that educating mothers on non-cavitated caries lesions requires not only active, visual instruction (e.g., ‘Lift the Lips’ self-examination techniques) but also periodic reinforcement during routine prenatal and pediatric visits to ensure long-term information retention and timely intervention.

The educational intervention should be interpreted in the context of the baseline findings observed in this study. Despite the relatively high educational level of the sample, important knowledge gaps persisted regarding several key aspects of maternal and child oral health, including early oral microbial colonization, the relationship between oral health and pregnancy outcomes, the appropriate use of fluoride, the recommended timing of the first dental visit, and the recognition of early caries lesions. These deficiencies highlight the need for preventive educational strategies during pregnancy and provide a strong rationale for evaluating the effectiveness of oral health interventions in this population.

The results of the present study demonstrated that a brief written educational intervention produced a statistically significant improvement in oral health knowledge (*p* = 0.018). Furthermore, a large proportion of participants improved their scores (*n* = 69) while 14 women showed no change. These findings suggest that even a low-intensity intervention based solely on written educational materials can positively influence oral health literacy during pregnancy.

Our findings are consistent with previous studies reporting significant improvements in oral health knowledge following educational interventions. Bansal et al. [13], for example, observed a significant increase in knowledge scores after the delivery of an educational leaflet, with median scores increasing from 4 to 7 points (*p* < 0.001). Similarly, Vilella et al. [33] and Cárdenas et al. [48] reported improvements in both knowledge acquisition and knowledge retention following oral health education programs. Collectively, these studies indicate that educational interventions during pregnancy consistently improve oral health knowledge, regardless of the format employed. This reinforces the utility of low-intensity educational strategies in primary care settings. A systematic review by Von Helde et al. [49] confirmed that educational tools integrated into prenatal oral health programs effectively reduce maternal plaque biofilm, dental caries, and periodontitis, while offering a high certainty of evidence for preventing ECC in offspring. However, expanding beyond passive printed materials may yield superior long-term clinical outcomes. For instance, Chen et al. [50] demonstrated that a multi-channel intervention structured around the Health Belief Model, incorporating video calls, online lectures, and digital follow-ups, achieved sustained enhancements in maternal self-efficacy (*p* < 0.001), oral health behaviors (*p* < 0.05), Plaque Index (*p* = 0.001), and Gingival Index (*p* = 0.021). Similarly, a randomized controlled trial by Singh et al. [51] established that culturally tailored animated video education produced significantly lower Plaque Index (*p* = 0.011) and Gingival Index scores (*p* < 0.001) at 6 months compared to conventional chairside model-based instruction. Incorporating dynamic visual or digital tools alongside concise printed leaflets could thus optimize both information retention and active behavioral modification.

However, the optimal method for delivering oral health information during pregnancy remains a subject of ongoing debate, as the magnitude of improvement depends heavily on the design and intensity of the intervention. While brief written interventions, such as the one used in the present study, generally produce modest gains, more comprehensive programs report larger effects [33]. Evidence shows that structured face-to-face counselling, direct verbal communication, and audiovisual materials, such as video-based tools, are particularly beneficial, especially for women with lower literacy levels [11,38,48,49]. Similarly, active strategies like motivational interviewing have been shown to outperform passive digital education (e.g., WhatsApp-based messaging) in improving both knowledge and oral hygiene outcomes [52]. Furthermore, interventions grounded in behavioral frameworks, such as the Health Belief Model [50,53] and the Theory of Planned Behavior [54], achieve broader, more consistent results by addressing both knowledge gaps and underlying behavioral determinants [50]. Together, these findings support the view that educational strategies are most effective when combining structured formats with theory-based approaches tailored to the population’s needs.

Importantly, increases in knowledge do not always translate into behavioral change. Chawla et al. [38] reported significant improvements in knowledge and attitudes following an educational program, whereas oral hygiene practices remained largely unchanged. In contrast, more intensive interventions conducted by Bahri et al. [16], Lin et al. [55], and Mohammadkhah et al. [54] achieved significant improvements in behaviors that persisted during follow-up. These findings suggest that changing health-related behaviors requires more than information alone and may depend on reinforcement strategies, self-efficacy, motivation, social support, and access to dental care services.

Although the impact of written educational interventions may be more limited than that of intensive face-to-face programs, the present findings demonstrate that even a simple informational leaflet can produce measurable improvements in oral health knowledge during pregnancy. Given their low cost, ease of implementation, and potential for broad dissemination, written educational materials may represent a valuable component of prenatal oral health promotion programs.

The findings of this study indicate that oral health education should be incorporated as a routine component of prenatal care. Important knowledge deficits persisted despite the relatively high educational level of the sample, suggesting that oral health promotion should be offered universally rather than restricted to specific risk groups. Pregnancy represents a unique opportunity to deliver preventive messages, and collaboration between midwives, obstetricians, primary care professionals, and dental practitioners may help improve maternal oral health literacy and support healthier behaviors for both mothers and their children. Together, these observations indicate that sociodemographic factors significantly influence the extent to which women benefit from educational programs. A one-size-fits-all approach is therefore insufficient, and educational strategies should be adapted to the literacy level and informational needs of the target population.

It must be acknowledged as a methodological limitation that the data collection instrument was not subjected to a full psychometric validation process. Although content validity was initially supported by adapting items from previously validated studies and aligning them with international maternal–child oral health guidelines, the synthesis of items from various sources meant that construct validity and reliability (internal consistency) were not formally evaluated for the unified questionnaire. Nevertheless, preliminary feasibility and clarity were confirmed through pilot testing, demonstrating the instrument’s practical functionality for the scope of this study. Future research should address this limitation by performing a comprehensive psychometric assessment.

Several additional limitations inherent to the study design should also be acknowledged. As a non-randomized, single-group pre–post study without a control group, the observed improvement in knowledge cannot be attributed exclusively to the educational intervention, since other factors, such as information received from healthcare professionals during the hospital stay, prior exposure to the questionnaire (testing effect), or regression to the mean, may have contributed to the changes observed. Furthermore, only 130 of the 229 women who completed the baseline assessment (56.77%) completed the post-intervention questionnaire. This loss to follow-up may have introduced attrition bias, as women who completed both assessments may have differed from those who did not in terms of motivation, educational level, or baseline knowledge, potentially leading to an overestimation of the effect of the intervention and limiting the generalizability of the findings. Therefore, these results should be interpreted with caution, and future randomized controlled trials including a control group, longer follow-up periods, and strategies to minimize attrition are needed to confirm them.

## 5. Conclusions

Pregnant women in this study demonstrated a moderate level of oral health knowledge; however, important gaps persisted regarding several key aspects of maternal and child oral health, including maternal impact on early infant oral microbial establishment, the relationship between oral health and pregnancy outcomes, fluoride use, early caries detection, and the recommended timing of the first dental visit. Educational attainment emerged as one of the main factors associated with higher knowledge levels, highlighting the influence of health literacy on oral health awareness during pregnancy.

The brief written educational intervention produced a statistically significant improvement in oral health knowledge. While these findings reflect a modest overall impact, they suggest that even low-intensity interventions can contribute to enhancing maternal oral health literacy. Integrating structured oral health education into routine antenatal care and strengthening collaboration between dental and prenatal healthcare professionals may help address existing knowledge gaps and promote healthier behaviors for both mothers and their children.

## Figures and Tables

**Table 1:** Structure and content of the educational leaflet.

Section	Core Topics Addressed
For Mothers	Common gestational oral diseases (gingivitis, periodontitis, dental caries, and erosion). Impact of maternal oral health on pregnancy and fetal development. Oral hygiene guidelines and recommendations for visiting the dentist during pregnancy.
For Your Baby	Primary teeth eruption chronology. Prevention of early childhood caries: healthy diet and timely cessation of pacifiers/teats. Infant oral hygiene protocols, including the use of fluoridated toothpaste. Preventive habits (e.g., avoiding spoon-sharing or cross-contamination). Recommendations for the child’s first visit to the paediatric dentist.

**Table 2:** Demographic data in completed initial questionnaires.

Study Variable		Sample (*n*)	Percentage
Birthplace	Spain	185	81.86%
	Abroad	41	18.14%
Marital status	In a relationship	211	92.54%
	Not in a relationship	17	7.46%
Previous children	Yes	108	47.37%
	No	120	52.63%
Level of education	College students	127	56.19%
	Non-college students	99	43.81%
Employment status	Paid work	164	72.89%
	Unpaid work	61	27.11%
Did you have any health problems during your pregnancy?	Yes	56	25.11%
	No	167	74.89%
Prenatal care	Public	146	64.32%
	Public + private	81	35.65%
Baby’s sex	Female	74	43.79%
	Male	95	56.21%
Do any of your other children have cavities?	Yes	13	12.04%
	No	91	84.26%
	I don’t know	4	3.70%

Percentages are calculated based on valid responses for each item (*n* = 223 to 228), as minor item non-response occurred across certain sociodemographic variables (N = 229 total baseline sample).

## Data Availability

The data supporting the findings of this study, including the Supplementary Materials submitted for editorial review, are available from the corresponding author upon reasonable request.

## Supplementary Materials

The following supporting information can be downloaded at https://www.mdpi.com/article/10.3390/dj14100683/s1. File S1: Baseline questionnaire Spanish version. File S2. Baseline questionnaire English version, File S3: Descriptive analysis of knowledge responses. Pregnant and puerperal women. Initial surveys.

## Abbreviations

The following abbreviations are used in this manuscript:

WHO	World Health Organization
AAPD	American Academy of Pediatric Dentistry
EAPD	European Academy of Paediatric Dentistry
ALOP	Asociación Latinoamericana de Odontopediatría
SEOP	Sociedad Española de Odontopediatría

## References

[1] Jain K., Kaur H. (2015). Prevalence of oral lesions and measurement of salivary pH in the different trimesters of pregnancy. Singap. Med. J..

[2] Hartnett E., Haber J., Krainovich-Miller B., Bella A., Vasilyeva A., Lange Kessler J. (2016). Oral health in pregnancy. J. Obstet. Gynecol. Neonatal Nurs..

[3] Asociación Latinoamericana de Odontopediatría (2016). Guía de Salud Bucal En Gestantes Para El Equipo Multidisciplinario.

[4] Zi M.Y.H., Longo P.L., Bueno-Silva B., Mayer M.P.A. (2015). Mechanisms involved in the association between periodontitis and complications in pregnancy. Front. Public Health.

[5] Kandan P.M., Menaga V., Kumar R.R.R. (2011). Oral health in pregnancy (guidelines to gynaecologists, general physicians & oral health care providers). J. Pak. Med. Assoc..

[6] Pitts N., Baez R., Diaz-Guallory C., Donly K.J., Feldens C.A., McGrath C., Phantumvanit P., Seow W.K., Sharkov N., Tinanoff N. (2019). Early childhood caries: IAPD Bangkok declaration. Int. J. Paediatr. Dent..

[7] Phantumvanit P., Makino Y., Ogawa H., Rugg-Gunn A., Moynihan P., Petersen P.E., Evans W., Feldens C.A., Lo E., Khoshnevisan M.H. (2018). WHO global consultation on public health intervention against early childhood caries. Community Dent. Oral Epidemiol..

[8] Touriño S., Suárez-Cotelo M.C., Núñez-Iglesias M.J., Domínguez-Martís E.M., Mosteiro-Miguéns D.G., López-Ares D., Novío S. (2021). Knowledge, attitudes, and practices of Spanish midwives and midwifery students toward oral healthcare during pregnancy. Int. J. Environ. Res. Public Health.

[9] Rigo L., Dalazen J., Garbin R.R. (2016). Impact of dental orientation given to mothers during pregnancy on oral health of their children. Einstein.

[10] Aznar-Marín A., Casaña-Ruiz M.A., Perales-Marín A., Catalá-Pizarro M. (2026). Maternal knowledge of oral health during pregnancy and early childhood: A systematic review. J. Clin. Exp. Dent..

[11] Gambhir R.S., Nirola A., Gupta T., Sekhon T.S., Anand S. (2015). Oral health knowledge and awareness among pregnant women in India: A systematic review. J. Indian Soc. Periodontol..

[12] Des Jarlais D.C., Lyles C., Crepaz N., TREND Group (2004). Improving the reporting quality of nonrandomized evaluations of behavioral and public health interventions: The TREND statement. Am. J. Public Health.

[13] Bansal K., Kharbanda O.P., Sharma J.R., Sood M., Priya H., Kriplani A. (2019). Effectiveness of an integrated perinatal oral health assessment and promotion program on the knowledge in Indian pregnant women. J. Indian Soc. Pedod. Prev. Dent..

[14] Kotha S.B., Alabdulaali R.A., Dahy W.A., Alkhaibari Y.M., Albaraki A.M., Alghanim A.A. (2018). The influence of oral health knowledge on parental practices among the Saudi parents of children aged 2–6 years in Riyadh city, Saudi Arabia. J. Int. Soc. Prev. Community Dent..

[15] Chala S., Houzmali S., Abouqal R., Abdallaoui F. (2018). Knowledge, attitudes and self-reported practices toward children oral health among mother’s attending maternal and child’s units, Salé, Morocco. BMC Public Health.

[16] Bahri N., Tohidinik H.R., Bahri N., Iliati H.R., Moshki M., Darabi F. (2015). Educational intervention to improve oral health beliefs and behaviors during pregnancy: A randomized-controlled trial. J. Egypt. Public Health Assoc..

[17] Dhull K.S., Dutta B., Devraj I.M., Samir P.V. (2018). Knowledge, attitude, and practice of mothers towards infant oral healthcare. Int. J. Clin. Pediatr. Dent..

[18] American Academy of Pediatric Dentistry (2025). Perinatal and infant oral health care. The Reference Manual of Pediatric Dentistry.

[19] European Academy of Paediatric Dentistry A Guide to Oral Health for Prospective Mothers and Their Infants. https://www.eapd.eu/posts/category/a-guide-to-oral-health-for-prospective-mothers-and-their-infants.

[20] Sociedad Española de Odontopediatría Embarazo y Salud Bucodental. https://www.odontologiapediatrica.com/.

[21] Núñez J., Moya P., Monsalves M.J., Landaeta S. (2013). Nivel de conocimiento de salud oral y utilización de GES odontológico en puérperas atendidas en una clínica privada, Santiago, Chile. Int. J. Odontostomatol..

[22] Aguilar-Cordero M.J., Mur-Villar N., García-García I., Castro-Sánchez A.M., Rivas-Campos A., Cortez-Molina A. (2018). Nivel de conocimiento sobre salud oral de pacientes gestantes: Estudio descriptivo. J. Negat. No Posit. Results.

[23] Seyyedi S.A., Asadollahi A., Dalirsani Z., Abdollahzadegan Z., Rezaei M. (2023). Knowledge and attitude of pregnant women in Urmia, Iran, about oral health care during pregnancy. Clin. Exp. Dent. Res..

[24] Abiola A.A., Olayinka A.O., Mathilda B.O., Ogunbiyi O.O., Olubunmi O.O. (2011). A survey of the oral health knowledge and practices of pregnant women in a Nigerian teaching hospital. Afr. J. Med. Med. Sci..

[25] Barbieri W., Peres S.V., Pereira C.D.B., Peres Neto J., Sousa M.L.R., Cortellazzi K.L. (2018). Sociodemographic factors associated with pregnant women’s level of knowledge about oral health. Einstein.

[26] Desai J., Varkey I.M., Lad D., Ghule K.D., Mathew R., Gomes S. (2022). Knowledge and attitude about infant oral health: A paradox among pregnant women. J. Contemp. Dent. Pract..

[27] Chawłowska E., Karasiewicz M., Lipiak A., Staszewski R., Cofta M., Biskupska M., Giernaś B., Zawiejska A. (2022). Oral health behaviours, knowledge, and literacy of expectant mothers: A cross-sectional study among maternity ward patients. Int. J. Environ. Res. Public Health.

[28] Wassihun B., Ayinalem A., Beyene K. (2022). Knowledge of oral health during pregnancy and associated factors among pregnant mothers attending antenatal care at South Omo Zone public hospitals, Southern Ethiopia, 2021. PLoS ONE.

[29] Niazi S., Eusufzai S.Z., Saddki N. (2023). Predictors of oral health literacy in pregnant women. Health Care Women Int..

[30] Llena C., Nakdali T., Sanz J.L., Forner L. (2019). Oral health knowledge and related factors among pregnant women attending to a primary care center in Spain. Int. J. Environ. Res. Public Health.

[31] Bhandari N., Chacko L.K., Shenoy R.P., Kalladka P.K. (2025). Pregnant mothers knowledge, attitude, and practice toward oral health: A cross-sectional study. J. Int. Oral Health.

[32] Motta M.C., Clemente G.O.S., Cavalcanti Y.W., Martins M.L., Fonseca-Gonçalves A. (2026). Knowledge, attitudes, and practices of pregnant women regarding maternal and child oral health: Questionnaire development, psychometric properties, and associated factors. Gen. Dent..

[33] Vilella K.D., Fraiz F.C., Benelli E.M., Assunção L.R.D. (2017). Oral health literacy and retention of health information among pregnant women: A randomised controlled trial. Oral Health Prev. Dent..

[34] Hammad O., Binsaad S., Gasim R., Jambi S., Haidary R., Afandi A., Abu-Hammad S., Odeh N. (2018). Assessing expectant mothers’ knowledge and beliefs about oral healthcare during infancy and pregnancy: A cross sectional survey in Saudi Arabia. Pesqui. Bras. Odontopediatr. Clín. Integr..

[35] Hom J.M., Lee J.Y., Divaris K., Baker A.D., Vann W.F. (2012). Oral health literacy and knowledge among patients who are pregnant for the first time. J. Am. Dent. Assoc..

[36] Cagetti M.G., Salerno C., Ionescu A.C., La Rocca S., Camoni N., Cirio S., Campus G. (2024). Knowledge and attitudes on oral health of women during pregnancy and their children: An online survey. BMC Oral Health.

[37] Lakshmi S.V., Srilatha A., Satyanarayana D., Reddy L.S., Chalapathi S., Meenakshi S. (2020). Oral health knowledge among a cohort of pregnant women in South India: A questionnaire survey. J. Fam. Med. Prim. Care.

[38] Chawla R.M., Mitra P., Shetiya S.H., Agarwal D.R., Narayana D.S., Bomble N.A. (2017). Knowledge, attitude, and practice of pregnant women regarding oral health status and treatment needs following oral health education in Pune district of Maharashtra: A longitudinal hospital-based study. J. Contemp. Dent. Pract..

[39] Gaszyńska E., Klepacz-Szewczyk J., Trafalska E., Garus-Pakowska A., Szatko F. (2015). Dental awareness and oral health of pregnant women in Poland. Int. J. Occup. Med. Environ. Health.

[40] Gaffar B., El Tantawi M., Al-Ansari A., AlAgl A. (2016). Association between oral health knowledge and practices of Saudi pregnant women in Dammam, Saudi Arabia. East. Mediterr. Health J..

[41] Gavic L., Maretic A., Putica S., Tadin A. (2022). Attitudes and knowledge of pregnant women about oral health. J. Educ. Health Promot..

[42] George A., Johnson M., Blinkhorn A., Ajwani S., Bhole S., Yeo A.E., Ellis S. (2013). The oral health status, practices and knowledge of pregnant women in South-western Sydney. Aust. Dent. J..

[43] Montoya-Carralero J.M., Ávila-Villasmil R., Sánchez-Pérez A., Jornet-García A., Terrer-Alonso E., Moya-Villaescusa M.J. (2024). Relationship between periodontal disease and preterm birth. A systematic review and meta-analysis. Med. Oral Patol. Oral Cir. Bucal.

[44] Rosier B., de Jager M., Zaura E., Krom B. (2014). Historical and contemporary hypotheses on the development of oral diseases: Are we there yet? Front. Cell. Infect. Microbiol..

[45] Marsh P. (2017). Ecological events in oral health and disease: New opportunities for prevention and disease control?. J. Calif. Dent. Assoc..

[46] Boggess K.A., Urlaub D.M., Moos M.K., Polinkovsky M., El-Khorazaty J., Lorenz C. (2011). Knowledge and beliefs regarding oral health among pregnant women. J. Am. Dent. Assoc..

[47] Angarita-Díaz M.D.P., Durán-Arismendy E., Cabrera-Arango C., Vásquez-Aldana D., Bautista-Parra V., Laguna-Moreno J., Mondragón-López W. (2024). Enhancing Knowledge, Attitudes, and Practices Related to Dental Caries in Mothers and Caregivers of Children through a Neuroeducational Strategy. BMC Oral Health.

[48] Cardenas L.M., Ross D.D. (2010). Effects of an oral health education program for pregnant women. J. Tenn. Dent. Assoc..

[49] Von Helde N.M., Martins M.L., da Costa Motta M., de Souza T.F., Magno M.B., Maia L.C., Fonseca-Gonçalves A. (2024). Are educational tools in oral health programs for pregnant women effective in improving the oral status of mothers and babies? A systematic review. Evid. Based Dent..

[50] Chen P., Li M., Zhang Q., Qiu M., Yu X. (2026). Effects of an oral health management program during pregnancy: A randomized controlled trial. Oral Dis..

[51] Singh P., Gupta V.K., Deo S., Rajput A., Khot A.J.P. (2026). Effectiveness of customized animated video-based oral health education compared with conventional model-based education on oral hygiene status and practices among pregnant women attending a tertiary care hospital: A single-blind randomized controlled trial. Spec. Care Dent..

[52] Basheer N.A., Jodalli P., Gowdar I.M., Almalki S.A. (2024). Effectiveness of motivational interviewing and cross platform messaging application in improving oral health knowledge, attitude and behaviours among pregnant women—A randomized controlled trial. F1000Research.

[53] Jeihooni A.K., Jamshidi H., Kashfi S.M., Avand A., Khiyali Z. (2017). The effect of health education program based on health belief model on oral health behaviors in pregnant women of Fasa City, Fars Province, South of Iran. J. Int. Soc. Prev. Community Dent..

[54] Mohammadkhah F., Amirhajelu R.M., Bakhtiar M., Salemi S.A., Kevenjan M., Jeihooni A.K. (2023). The effect of training intervention based on the theory of planned behavior on oral and dental health behaviors in pregnant women. BMC Oral Health.

[55] Lin D.L., Harrison R., Aleksejuniene J. (2011). Can a prenatal dental public health program make a difference?. J. Can. Dent. Assoc..

