# Supplementary material for: Knowledge of Oral Health in Pregnant and Puerperal Women: Effect of a Short Educational Intervention
Source: Dent J (Basel). 2026 Oct 19;14(10):683. doi: 10.3390/dj14100683 (PMC13703064; doi:10.3390/dj14100683)
Supplement: Supplementary file 1 [file dentistry-14-00683-s001.zip › dentistry-4515028-supplementary.pdf]

Supplementary File S1: *Oral Health Surveys for Pregnant Women (Spanish version).*

Encuestas de salud oral para mujeres embarazadas

Estimado Sr/Sra.:

Estamos muy interesados en conocer su opinión sobre la salud dental de los niños y por ello realizamos estas encuestas.

Sus respuestas son totalmente anónimas y todo lo que diga será tratado de forma absolutamente confidencial.

Es importante que conteste a todas las preguntas de la encuesta y en caso de duda puede consultar a la persona que la entrevista.

Le agradecemos sinceramente su colaboración.

Servicio de Ginecología y Obstetricia

Datos demográficos

1. ¿Qué edad tiene?
   ………………………………
2. Lugar de nacimiento:
   a. España
   b. Europa
   c. América del norte
   d. América del sur
   e. Asia
   f. África
3. Nivel de estudios:
   a. Sin estudios
   b. Primarios
   c. Bachillerato o F.P.
   d. Universitarios
4. Situación laboral:
   a. Paro
   b. Trabajo en casa (ama de casa)
   c. Trabajo fuera de casa
5. Estado civil:
   a. Pareja estable
   b. Casada
   c. Soltera
   d. Divorciada
6. A parte de este embarazo ¿tiene usted más hijos?
   a. No
   b. Sí
7. ¿Alguno de sus otros hijos tienen caries?
   a. Si
   b. No

   No lo sé

8. ¿Durante el embarazo tuvo algún problema de salud? (en caso afirmativo especifique cual)
   a. Si ……………………………
   b. No
9. Seguimiento del embarazo:
   a. Centro sanitario público
   b. Centro sanitario privado
   c. Ambos
10. Duración del embarazo (semanas)

11. Tipo de parto
    a. Vaginal

b. Vaginal con vacuum o Forceps
c. Cesárea

12. Peso del recién nacido

    ----

13. Sexo del bebé
    a. Femenino
    b. Masculino
14. Estado de salud del bebé:
    a. Lo tiene usted en la habitación
    b. Ingresado en pediatría
    c. Ingresado en UCIN

Conocimiento salud oral

1. ¿Sabía que durante el embarazo existen cambios hormonales que afectan al estado de sus encías?
   a. Si, me informó mi médico/matrona/dentista
   b. Sí, me informó familiar/amigo/conocido
   c. No, nunca me informaron
2. ¿Puede el cepillado diario prevenir los problemas de las encías y caries?
   a. Si
   b. No
   c. No lo sé
3. ¿La caries está causada por bacterias?
   a. Si
   b. No
   c. No lo sé
4. ¿Piensa que sus problemas dentales pueden afectar a la salud del feto/ futuro hijo?
   a. Si
   b. No
   c. No lo sé
5. ¿Cree que se pueden transmitir de padres a hijos bacterias que producen caries?
   a. Si
   b. No
   c. No lo sé
6. ¿Pueden los problemas de los dientes de leche afectar a los dientes de adulto?
   a. Si
   b. No
   c. No lo sé
7. ¿Cree que puede ayudar a prevenir las caries de su hijo/a?
   a. Si
   b. No
   c. No lo sé
8. ¿Sabe cuándo debe comenzar a limpiar la boca del bebé?

a. Desde el nacimiento
b. Con la erupción del primer diente
c. A partir 2-3 años
d. No lo sé

9. Acostar al niño con un biberón que contenga leche o zumo se asocia a caries incluso en niños de 2 o 3 años
   a. Si
   b. No
   c. No lo sé
10. ¿Es cierto que se desaconseja compartir utensilios con el bebé, limpiar el chupete con saliva o besar en la boca al bebé?
    a. Si
    b. No
    c. No lo sé
11. ¿Cree que el flúor en la pasta de dientes previene la caries?
    a. Si
    b. No
    c. No lo sé
12. ¿Se puede usar dentífrico con flúor en niños menores de 3 años?
    a. Si
    b. No
    c. No lo sé
13. ¿Cuándo cree que debe ser la primera visita al dentista?
    a. A los 12 meses
    b. Cuando tuviera todos los dientes de leche
    c. A partir 6 años
    d. Cuando tenga sospecha de caries
14. ¿La primera señal de caries suele ser una mancha blanca?
    a. Si
    b. No
    c. No lo sé

Actitudes y prácticas de salud oral

1. Frecuencia del cepillado dental:
   a. Nunca
   b. Raramente/ Ocasionalmente
   c. Diariamente: 1 v/d 2 v/d 3 v/d o más
2. ¿Cuál es su experiencia personal en el dentista?
   a. Hace años que no voy al dentista
   b. Voy regularmente todos los años, aunque no tenga molestias
   c. Voy cuando tengo molestias
3. Durante el embarazo ¿Acudió al dentista?
   a. Si

    b. No
4. ¿Le preguntó a su dentista, médico o matrona acerca de las pautas de salud oral que debía seguir durante el embarazo?
    a. Si
    b. No
5. ¿Cree que es necesario vigilar la salud de los dientes de leche?
    a. Si
    b. No
    c. No lo sé
6. Tipo de alimentación que desea dar/da a su bebé:
    a. Pecho
    b. Biberón +leche materna
    c. Biberón + Fórmula de leche
    d. Mixta
7. Hasta que edad quiere darle el pecho:
    a. 1 mes
    b. 3 meses
    c. 6 meses
    d. 12 meses
    e. Hasta que pueda
8. ¿Cómo considera que es la alimentación de sus otros hijos?:
    a. Blanda
    b. Semi-sólida
    c. Fibrosa y "dura"
9. Con qué frecuencia toman sus hijos dulces (chocolates, galletas, chucherías, zumos, batidos, cereales, yogures bebidos, natillas…)
    a. 1 vez al día
    b. 2 veces al día
    c. >3 veces al día
10. Usan pasta de dientes fluorada:
    a. Si
    b. No

Formación salud oral

1. ¿Sabía que durante el embarazo existen cambios hormonales que afectan al estado de sus encías?
    a. Si, me informó mi médico/matrona/dentista
    b. Sí, me informó familiar/amigo/conocido
    c. No, nunca me informaron
2. ¿Ha recibido información acerca de la importancia de cuidar su boca durante el embarazo?
    a. Si
    b. No
3. En caso afirmativo, ¿Por parte de quién?

a. Médico de cabecera
b. Ginecólogo
c. Matrona
d. Dentista
e. Familiar/amigo/conocido

4. ¿Ha recibido información acerca de salud oral y prevención de caries en el bebé?
    a. Si
    b. No
5. En caso afirmativo, ¿Por parte de quién?
    a. Ginecólogo
    b. Pediatra
    c. Médico de cabecera
    d. Matrona
    e. Dentista
    f. Otros ……………………….

HOJA DE INFORMACIÓN AL PACIENTE ADULTO

Versión: 1.1	Fecha de la versión: 4/11/2021
Investigador Principal: Beatriz Marcos Puig. Departamento de Ginecología y obstetricia. beatrizmarcosp@gmail.com	
CENTRO: Hospital Universitario y Politécnico de la Fe	
Título del proyecto de investigación<br>Conocimiento de salud oral en mujeres embarazadas o puérperas y personal sanitario	

1. **INTRODUCCIÓN:**

Nos dirigimos a usted para informarle sobre un estudio en el que se le invita a participar. El estudio ha sido aprobado por el Comité de Ética de la Investigación con medicamentos del Hospital Universitario y Politécnico La Fe (CEIm La Fe).

Nuestra intención es tan sólo que usted reciba la información correcta y suficiente para que pueda evaluar y juzgar si quiere o no participar en este estudio. Para ello lea esta hoja informativa con atención y nosotros le aclararemos las dudas que le puedan surgir después de la explicación. Además, puede consultar con las personas que considere oportunas.

2. **PARTICIPACIÓN VOLUNTARIA:**

Debe saber que su participación en este estudio es voluntaria y que puede decidir no participar y retirar el consentimiento en cualquier momento, sin que por ello se altere la relación con su médico ni se produzca perjuicio alguno en su tratamiento.

3. **DESCRIPCIÓN GENERAL DEL ESTUDIO:**

Consiste en la realización de encuestas dirigidas a mujeres embarazadas y puérpera atendidas en el Hospital Universitari i Politècnic La Fe; y personal sanitario (médicos y enfermeras) del mismo centro.

Se pretende obtener datos acerca de la educación en salud oral en embarazadas y mujeres puérperas y del personal sanitario, que permitan analizar y evaluar la efectividad de la educación y de las medidas preventivas. .

Las encuestas se realizarán de forma presencial en el servicio de ginecología y obstetricia del hospital, vía telefónica y telemática a través de un e-mail. En caso de duda, esta se resolverá poniéndose en contacto con los investigadores del estudio. Se estima unos 15 minutos para la cumplimentación de la misma.

4. BENEFICIOS Y RIESGOS DERIVADOS DE SU PARTICIPACIÓN EN EL ESTUDIO:

No existen riesgos asociados a la participación en este estudio, ya que se deben cumplimentar unas encuestas, por lo que no tendrá consecuencias sobre la salud de las personas.

Debe saber que su participación en este estudio puede no aportarle beneficios directos.

5. Nº DE URGENCIA PARA PROBLEMAS DEL ESTUDIO:

En caso de que desee formular preguntas acerca del estudio o daños relacionados con el mismo, puede contactar con el médico del estudio, el Dña Alicia Aznar Marín, en el número de teléfono 625063066.

6. CONFIDENCIALIDAD:

El tratamiento, la comunicación y la cesión de los datos de carácter personal de todos los sujetos participantes se ajustará a lo dispuesto en la Ley Orgánica 3/2018, de 5 de diciembre de protección de datos y garantía de los derechos digitales y en el Reglamento (UE) 2016/679 del Parlamento europeo y del Consejo de 27 de abril de 2016 de Protección de Datos (RGPD) . De acuerdo a lo que establece la legislación mencionada, usted puede ejercer los derechos de acceso, modificación, oposición, cancelación de datos, limitar el tratamiento de datos que sean incorrectos y solicitar una copia o que se trasladen a un tercero (portabilidad) para lo cual deberá dirigirse al investigador principal del estudio o al/a la Delegado/a de Protección de Datos de la Institución, con quién podrá comunicarse a través del teléfono y/o dirección de correo electrónico: aliaznmar90@gmail.com.

Le recordamos que los datos no se pueden eliminar, aunque deje de participar en el estudio, para garantizar la validez de la investigación y cumplir con los deberes legales. Así mismo tiene derecho a dirigirse a la Agencia de Protección de Datos si no quedara satisfecho.

Tanto el Centro como el Promotor son responsables respectivamente del tratamiento de sus datos y se comprometen a cumplir con la normativa de protección de datos en vigor. Los datos recogidos para el estudio estarán identificados mediante un código, de manera que no se incluya información que pueda identificarle, y sólo su médico del estudio/colaboradores podrá relacionar dichos datos con usted y con su historia clínica. Por lo tanto, su identidad no será revelada a ninguna otra persona salvo a las autoridades sanitarias, cuando así lo requieran o en casos de urgencia médica. Los Comités de Ética de la Investigación con medicamentos, los representantes de la Autoridad Sanitaria en materia de inspección y el personal autorizado por el Promotor, únicamente podrán acceder para comprobar los datos personales, los procedimientos del estudio clínico y el cumplimiento de las normas de buena práctica clínica (siempre manteniendo la confidencialidad de la información).

El Investigador y el Promotor están obligados a conservar los datos recogidos para el estudio al menos hasta 25 años tras su finalización. Posteriormente, su información personal solo se conservará por el centro para el cuidado de su salud y por el promotor para otros fines de investigación científica si usted hubiera otorgado su consentimiento para ello, y si así lo permite la ley y requisitos éticos aplicables.

Sólo se tramitarán a terceros y a otros países los datos recogidos para el estudio, que en ningún caso contendrán información que le pueda identificar directamente, como nombre y apellidos, iniciales, dirección, nº de la seguridad social, etc... En el caso de que se produzca esta cesión, será para los mismos fines del estudio descrito y garantizando la confidencialidad como mínimo con el nivel de protección de la legislación vigente en nuestro país.

Si realizáramos transferencia de sus datos codificados fuera de la UE a las entidades de nuestro grupo, a prestadores de servicios o a investigadores científicos que colaboren con nosotros, los datos del participante quedarán protegidos con salvaguardas tales como contratos u otros mecanismos por las autoridades de protección de datos. Si el participante quiere saber más al respecto, puede contactar con el Delegado de Protección de Datos del promotor

7. COMPENSACIÓN ECONÓMICA:

El promotor del estudio es el responsable de gestionar la financiación del mismo, por lo que su participación en éste no le supondrá ningún gasto.

8. OTRA INFORMACIÓN RELEVANTE:

Cualquier nueva información que se descubra durante su participación y que pueda afectar a su disposición para participar en el estudio, le será comunicada por su médico lo antes posible.

Si usted decide retirar el consentimiento para participar en este estudio, no se añadirá ningún dato nuevo a la base de datos y, en caso de que se hubieran recogido muestras, puede exigir la destrucción de todas las muestras identificables, previamente obtenidas, para evitar la realización de nuevos análisis.

También debe saber que puede ser excluido del estudio si el promotor o los investigadores del mismo lo consideran oportuno, ya sea por motivos de seguridad o porque consideren que usted no está cumpliendo con los procedimientos establecidos. En cualquiera de los casos, usted recibirá una explicación adecuada del motivo por el que se ha decidido su retirada del estudio.

El promotor podrá suspender el estudio siempre y cuando sea por alguno de los supuestos contemplados en la legislación vigente.

Al firmar la hoja de consentimiento adjunta, se compromete a cumplir con los procedimientos del estudio que se le han expuesto. Cuando acabe su participación recibirá el mejor tratamiento disponible y que su médico considere el más adecuado para su enfermedad.

CONSENTIMIENTO POR ESCRITO

Título del estudio: Conocimiento de salud oral en mujeres embarazadas o puérperas y personal sanitario

Yo, *(nombre y apellidos).*

- He leído la hoja de información que se me ha entregado.
- He podido hacer preguntas sobre el estudio.
- He recibido suficiente información sobre el estudio.
- He hablado con: Alicia Aznar Marín
- Comprendo que mi participación es voluntaria.
- Recibiré una copia firmada y fechada de este documento de consentimiento informado.

Comprendo que puedo retirarme del estudio:

1º Cuando quiera

2º Sin tener que dar explicaciones.

3º Sin que esto repercuta en mis cuidados médicos.

Presto libremente mi conformidad para participar en el estudio.

Firma del Participante ***Firma del Investigador***

Nombre: ________________________ ***Nombre:*** ________________________

Fecha: ____/____/____ *Fecha:* ____/____/____

Deseo que me comuniquen la información derivada de la investigación que pueda ser relevante para mí salud: ☐ **Sí** ☐ **No**

Firma del representante ___________________________ Fecha: ____/____/____ *(Nombre, firma y fecha de puño y letra del representante legal)*

Firma del investigador: ___________________________ Fecha: ____/____/____

Deseo que me comuniquen la información derivada de las pruebas genéticas realizadas (únicamente para aquellos estudios que incluyan este tipo de pruebas, siempre que estén validadas y que puedan tener relevancia para la salud del paciente): ☐ **Sí** ☐ **No**

Firma del representante ___________________________ Fecha: ____/____/____ *(Nombre, firma y fecha de puño y letra del representante legal)*

Firma del investigador: ___________________________ Fecha: ____/____/____

Supplementary File S2:*Oral Health Surveys for Pregnant Women (English version)*

Oral Health Surveys for Pregnant Women

Dear Sir/Madam:

We are very interested in learning about your views regarding children's oral health, and this is why we are conducting these surveys.

Your answers are completely anonymous, and everything you say will be treated as strictly confidential.

It is important that you answer all the questions in the survey. If you have any doubts, you may ask the person conducting the interview.

We sincerely appreciate your cooperation. Department of Obstetrics and Gynecology

Demographic Information

1. How old are you?

…………………………………

2. Place of birth:

    a. Spain
    b. Europe
    c. North America
    d. South America
    e. Asia
    f. Africa

3. Educational level:

    a. No formal education
    b. Primary education
    c. Upper secondary education or vocational training
    d. University education

4. Employment status:

    a. Unemployed
    b. Homemaker
    c. Employed outside the home

5. Marital status:

    a. In a stable relationship
    b. Married
    c. Single
    d. Divorced

6. Apart from this pregnancy, do you have any other children?

    a. No
    b. Yes

7. Do any of your other children have dental caries?

    a. Yes
    b. No
    c. I don't know

8. Did you have any health problems during pregnancy? (If yes, please specify.)

    a. Yes ………………………………
    b. No

9. Pregnancy follow-up:

    a. Public healthcare facility
    b. Private healthcare facility
    c. Both

10. Length of pregnancy (weeks)

………………………………

11. Type of delivery:
  a. Vaginal delivery
  b. Vaginal delivery with vacuum or forceps
  c. Cesarean section
12. Newborn's weight:
----
13. Baby's sex:
  a. Female
  b. Male
14. Baby's health status:
  a. Rooming-in with you
  b. Admitted to the pediatric ward
  c. Admitted to the Neonatal Intensive Care Unit (NICU)

Oral Health Knowledge

1. Did you know that hormonal changes occur during pregnancy that can affect the health of your gums?
  a. Yes, my doctor/midwife/dentist informed me
  b. Yes, a family member/friend/acquaintance informed me
  c. No, I had never been informed
2. Can daily toothbrushing prevent gum problems and dental caries?
  a. Yes
  b. No
  c. I don't know
3. Is dental caries caused by bacteria?
  a. Yes
  b. No
  c. I don't know
4. Do you think that your dental problems can affect the health of your fetus/future child?
  a. Yes
  b. No
  c. I don't know
5. Do you think that bacteria that cause dental caries can be transmitted from parents to children?
  a. Yes
  b. No
  c. I don't know
6. Can problems affecting primary teeth affect the permanent teeth?

a. Yes

b. No

c. I don't know

7. Do you think you can help prevent dental caries in your child?

a. Yes

b. No

c. I don't know

8. Do you know when you should start cleaning your baby's mouth?

a. From birth

b. When the first tooth erupts

c. From 2–3 years of age

d. I don't know

9. Putting a child to bed with a bottle containing milk or juice is associated with dental caries, even in children aged 2 or 3 years.

a. Yes

b. No

c. I don't know

10. Is it true that sharing utensils with the baby, cleaning the pacifier with saliva, or kissing the baby on the mouth is discouraged?

a. Yes

b. No

c. I don't know

11. Do you think that fluoride in toothpaste prevents dental caries?

a. Yes

b. No

c. I don't know

12. Can fluoride toothpaste be used in children under 3 years of age?

a. Yes

b. No

c. I don't know

13. When do you think your child should have their first dental visit?

a. At 12 months of age

b. When they have all their primary teeth

c. From 6 years of age

d. When dental caries is suspected

14. Is a white spot usually the first sign of dental caries?

a. Yes

b. No

c. I don't know

Oral Health Attitudes and Practices

1. Toothbrushing frequency:
   a. Never
   b. Rarely/occasionally
   c. Daily: once a day / twice a day / three times a day or more
2. What is your personal experience with the dentist?
   a. I have not been to the dentist for years
   b. I visit the dentist regularly every year, even if I have no problems
   c. I go to the dentist when I have problems
3. Did you visit the dentist during your pregnancy?
   a. Yes
   b. No
4. Did you ask your dentist, doctor, or midwife about the oral health recommendations you should follow during pregnancy?
   a. Yes
   b. No
5. Do you think it is necessary to monitor the health of primary teeth?
   a. Yes
   b. No
   c. I don't know
6. How do you plan to feed/are you feeding your baby?
   a. Breastfeeding
   b. Bottle-feeding + breast milk
   c. Bottle-feeding + infant formula
   d. Mixed feeding
7. How long would you like to breastfeed?
   a. 1 month
   b. 3 months
   c. 6 months
   d. 12 months
   e. As long as possible
8. How would you describe the diet of your other children?
   a. Soft

b. Semi-solid

c. Fibrous and "hard"

9. How often do your children consume sweets or sweet foods/drinks (chocolate, biscuits, candy, juices, milkshakes, cereals, drinkable yogurts, custard, etc.)?

a. Once a day

b. Twice a day

c. More than 3 times a day

10. Do they use fluoride toothpaste?

a. Yes

b. No

Oral Health Education

1. Did you know that hormonal changes occur during pregnancy that can affect the health of your gums?

a. Yes, my doctor/midwife/dentist informed me

b. Yes, a family member/friend/acquaintance informed me

c. No, I had never been informed

2. Have you received information about the importance of taking care of your mouth during pregnancy?

a. Yes

b. No

3. If yes, who provided you with this information?

a. General practitioner/family doctor

b. Obstetrician-gynecologist

c. Midwife

d. Dentist

e. Family member/friend/acquaintance

4. Have you received information about oral health and prevention of dental caries in babies?

a. Yes

b. No

5. If yes, who provided you with this information?

a. Obstetrician-gynecologist

b. Pediatrician

c. General practitioner/family doctor

d. Midwife

e. Dentist

f. Other ……………………….

ADULT PATIENT INFORMATION SHEET

Version: 1.1 Date of version: 4 November 2021

Principal Investigator: Beatriz Marcos Puig. Department of Obstetrics and Gynecology.

beatrizmarcosp@gmail.com

SITE: Hospital Universitario y Politécnico La Fe

Title of the research project

Oral Health Knowledge in Pregnant or Postpartum Women and Healthcare Professionals

1. INTRODUCTION:

We are providing you with information about a study in which you are being invited to participate. The study has been approved by the Research Ethics Committee for Medicinal Products of Hospital Universitario y Politécnico La Fe (CEIm La Fe).

Our sole purpose is to provide you with the correct and sufficient information so that you can decide whether or not you wish to participate in this study. Please read this information sheet carefully. We will clarify any questions you may have after the explanation.

You may also consult with anyone you consider appropriate.

2. VOLUNTARY PARTICIPATION:

You should know that your participation in this study is voluntary. You may decide not to participate or withdraw your consent at any time, without this affecting your relationship with your doctor or causing any disadvantage to your medical care.

3. GENERAL DESCRIPTION OF THE STUDY:

The study consists of surveys administered to pregnant and postpartum women receiving care at Hospital Universitario y Politécnico La Fe, as well as healthcare professionals (doctors and nurses) working at the same center.

The aim is to obtain information about oral health education among pregnant and postpartum women and healthcare professionals, in order to analyze and evaluate the effectiveness of education and preventive measures.

The surveys will be conducted in person in the hospital's Department of Obstetrics and Gynecology, by telephone, and electronically by email. If you have any questions, these will be addressed by

contacting the study investigators. The survey is expected to take approximately 15 minutes to complete.

4. BENEFITS AND RISKS ASSOCIATED WITH PARTICIPATION IN THE STUDY:

There are no risks associated with participation in this study, as participation consists of completing surveys and therefore will have no consequences for participants' health.

Please be aware that participation in this study may not provide you with any direct benefit.

5. EMERGENCY CONTACT FOR STUDY-RELATED PROBLEMS:

If you have any questions about the study or experience any study-related harm, you may contact the study physician, Dr. Alicia Aznar Marín, at 625063066.

6. CONFIDENTIALITY:

The processing, communication, and transfer of personal data of all participants will comply with Organic Law 3/2018 of 5 December on the Protection of Personal Data and Guarantee of Digital Rights, and with Regulation (EU) 2016/679 of the European Parliament and of the Council of 27 April 2016 on data protection (GDPR).

In accordance with the above legislation, you may exercise your rights of access, rectification, objection, erasure, restriction of processing of inaccurate data, and request a copy of your data or its transfer to a third party (data portability). To do so, you should contact the principal investigator of the study or the institution's Data Protection Officer, who can be contacted by telephone and/or email at: aliaznmar90@gmail.com.

We remind you that the data cannot be deleted, even if you withdraw from the study, in order to ensure the validity of the research and comply with legal obligations. You also have the right to contact the Spanish Data Protection Agency if you are not satisfied.

Both the Center and the Sponsor are responsible, respectively, for processing your data and undertake to comply with the data protection regulations in force. The data collected for the study will be identified by a code so that no information that could identify you is included. Only the study physician/collaborators will be able to link these data to you and your medical record. Therefore, your identity will not be disclosed to any other person except health authorities when required to do so or in the event of a medical emergency.

Research Ethics Committees for Medicinal Products, representatives of the Health Authority responsible for inspections, and personnel authorized by the Sponsor may access the data only to verify personal information, clinical study procedures, and compliance with Good Clinical Practice standards, while maintaining the confidentiality of the information at all times.

The Investigator and the Sponsor are required to retain the data collected for the study for at least 25 years after its completion. Subsequently, your personal information will only be retained by the center for your healthcare

and by the Sponsor for other scientific research purposes if you have given your consent for this and if permitted by applicable law and ethical requirements.

Only study data will be transferred to third parties and to other countries, and such data will never contain information that could directly identify you, such as your name and surname, initials, address, Social Security number, etc. If such a transfer takes place, it will be for the same purposes as those described in the study and confidentiality will be guaranteed with at least the level of protection required by the legislation in force in our country.

If we transfer your coded data outside the European Union to entities within our group, service providers, or scientific researchers collaborating with us, your data will be protected by safeguards such as contracts or other mechanisms required by data protection authorities. If you would like further information, you may contact the Sponsor's Data Protection Officer.

7. FINANCIAL COMPENSATION:

The Sponsor of the study is responsible for managing its funding; therefore, your participation in the study will not involve any cost to you.

8. OTHER RELEVANT INFORMATION:

Any new information discovered during your participation that may affect your willingness to continue participating in the study will be communicated to you by your doctor as soon as possible.

If you decide to withdraw your consent to participate in this study, no new data will be added to the database. If samples have been collected, you may request the destruction of all previously obtained identifiable samples to prevent further analyses.

You should also know that you may be withdrawn from the study if the Sponsor or the study investigators consider this appropriate, either for safety reasons or because they consider that you are not complying with the established procedures. In either case, you will receive an appropriate explanation of the reason for your withdrawal.

The Sponsor may suspend the study provided that this is done for any of the circumstances contemplated by current legislation.

By signing the attached consent form, you agree to comply with the study procedures explained to you. At the end of your participation, you will receive the best available treatment that your doctor considers most appropriate for your condition.

WRITTEN INFORMED CONSENT

Study title: Oral Health Knowledge in Pregnant or Postpartum Women and Healthcare Professionals

I, (full name).

• I have read the information sheet that has been provided to me.

• I have been able to ask questions about the study.

• I have received sufficient information about the study.

• I have spoken with: Alicia Aznar Marín

• I understand that my participation is voluntary.

• I will receive a signed and dated copy of this informed consent form.

I understand that I may withdraw from the study:

1. At any time.

2. Without having to give any explanation.

3. Without this affecting my medical care.

I freely consent to participate in the study.

Participant's signature

Name:

Date: ____/____/____

Investigator's signature

Name:

Date: ____/____/____

I would like to be informed of research findings that may be relevant to my health: ☐ Yes ☐ No

Representative's signature Date: ____/____/____ (Representative's full name, signature, and handwritten date)

Investigator's signature: Date: ____/____/____

I would like to be informed of the results of any genetic tests performed (only for studies that include this type of testing, provided that the tests are validated and may be relevant to the patient's health): ☐ Yes ☐ No

Representative's signature Date: ____/____/____ (Representative's full name, signature, and handwritten date)

Investigator's signature: Date: ____/____/____

Supplementary File S3: Descriptive analysis of knowledge responses. Pregnant and puerperal women. Initial surveys.

Variable Studied		Sample (n)	Percentage	No response (n, %)
Did you know that hormonal changes during pregnancy can affect the health of your gums?	Yes, midwife/doctor/dentist	141	62.11%	n=5 2.18%
	Yes, family member/friend	60	25.81%	
	I was never told	23	9.90%	
Can daily brushing prevent gum disease and cavities?	Yes	215	93.89%	-
	No	2	0.87%	
	I don't know	12	5.24%	
Are cavities caused by bacteria?	Yes	179	78.17%	n=1 0.44%
	No	1	0.44%	
	I don't know	48	20.96%	
Do you think your dental problems could affect the health of your unborn child?	Yes	124	54.15%	n=1 0.44%
	No	26	11.35%	
	I don't know	78	34.06%	
Do you think bacteria that cause cavities can be passed from parents to children?	Yes	89	38.86%	n=1 0.44%
	No	44	19.21%	
	I don't know	95	41.48%	
Can problems with baby teeth affect adult teeth?	Yes	158	69.00%	-
	No	7	3.06%	
	I don't know	64	27.95%	
Do you think you can help prevent your child from getting tooth decay?	Yes	228	99.56%	
	No	-		
	I don't know	1	0.44%	

Variable Studied		Sample (n)	Percentage	No response (n, %)
Do you know when you should start cleaning your baby's mouth?	From birth	53	23.14%	n=4 1.75%
	First tooth	99	43.23%	
	2–3 years	27	11.79%	
	I don't know	46	20.09%	
Letting your child fall asleep with a bottle containing milk or juice is associated with tooth decay, even in children as young as 2 or 1 years old	Yes	137	59.83%	n=6 2.62%
	No	5	2.18%	
	I don't know	81	35.37%	
Is it true that it's not recommended to share utensils with your baby, clean a pacifier with saliva, or kiss your baby on the mouth?	Yes	194	84.72%	n=5 2.18%
	No	6	2.62%	
	I don't know	24	10.48%	
Do you think the fluoride in toothpaste prevents tooth decay?	Yes	149	65.07%	n=7 3.06%
	No	19	8.73%	
	I don't know	53	23.14%	
Can fluoride toothpaste be used on children under 3 years of age?	Yes	56	24.45%	n=9 3.93%
	No	66	28.82%	
	I don't know	98	42.79%	
When do you think a child should have their first dental visit?	12 months	94	41.05%	n=13 5.68%
	All baby teeth	89	38.86%	
	6 years	21	9.17%	
	Suspected tooth decay	12	5.24%	
Is a white spot usually the first sign of tooth decay?	Yes	23	10.04%	n=6 2.62%
	No	38	16.59%	
	I don't know	162	70.74%	
